# Characteristic and optimization of the effective perspective images’ segmentation and mosaicking (EPISM) based holographic stereogram: an optical transfer function approach

**DOI:** 10.1038/s41598-018-22762-3

**Published:** 2018-03-14

**Authors:** Jian Su, Xingpeng Yan, Xiaoyu Jiang, Yingqing Huang, Yibei Chen, Teng Zhang

**Affiliations:** 1 Department of Information Engineering, Academy of Armored Forces Engineering, Beijing, 100072 China; 2 Academy of Armored Forces Engineering, Beijing, 100072 China

## Abstract

Based on our proposed method for holographic stereogram printing using effective perspective images’ segmentation and mosaicking (EPISM), we analyze the reconstructed wavefront errors, and establish the exit pupil function model of proposed printing system. To evaluate the imaging quality, the optical transfer function (OTF) of the holographic stereogram is modelled from the aspect of frequency response. The characteristic of the OTF with respect to the exit pupil size and the aberration are investigated in detail. We also consider the flipping effect in spatial domain. The optimization of hogel sizes, i.e., the sampling interval of original perspective images and the printing interval of synthetic effective perspective images, are given for the optimized reconstruction. Numerical simulations and optical experiments are implemented, and the results demonstrate the validity of our analysis, and the optimized parameters of hogel sizes can improve the imaging quality of full parallax holographic stereogram effectively.

## Introduction

In recent years, it has been a research hotspot to realize three-dimensional (3D) display by using holographic technology^1–7^. Holographic stereogram printing is a kind of holographic technology^8–12^. A series of perspective images of the 3D scene are first captured by a tracking camera or modeled by a computer with rendering techniques, then they are exposed and stored in small holographic elements called hogels^13^. With an appropriate illumination, each hogel will diffract a bundle of light rays, which are fractions of different perspective images, or just come from one perspective image within a certain viewing angle^14^. When the observer’s left and right eyes are located at different viewing points, he will receive the corresponding perspective images with parallax information, then the stereoscopic vision occurs. The parallax is changing when eyes are moving^15^.

Synthetic holographic stereogram was first proposed by DeBitetto^15^ and developed rapidly^16–28^. To achieve reconstructed images inside or outside the recording medium, there is the two-step method proposed by King *et al*.^29^, the infinite viewpoint camera method proposed by the researchers from Massachusetts Institute of Technology (MIT)^13,30^, the single-step Lippmann holographic stereogram method proposed by Yamaguchi’s group^31,32^, and the direct-write digital holography (DWDH) method reviewed by Bjelkhagen and Brotherton-Ratcliffe^11,18,33,34^. In the two-step method, the master hologram (H_1_ plate) should be optically recopied to the transfer hologram (H_2_ plate), so the process is relatively complex with twice exposures and it is difficult to make a large-size hologram since we can hardly achieve large-format collimating light during the second exposure. In the infinite viewpoint camera method, the distance between the sampling camera and the holographic recording medium is far enough, so the light arriving at the hogels can be considered as bundles of parallel light approximately and the perspective images are transformed into the parallax related images firstly. The resolution of reconstructed images equals to the number of hogels and it is relatively low, especially for a small size hologram. In the single-step Lippmann holographic stereogram method, the images for exposing are calculated by perspective projection. More specifically, the object points are projected to the position of liquid crystal display (LCD) panel, and the occlusion relation of object points in space should be considered and the hidden surfaces should be removed according to the viewer’s position. DWDH printers have been extensively reviewed by Bjelkhagen and Brotherton-Ratcliffe, and the core idea is the H_1_-H_2_ conversion, i.e., the image transformation from the camera film plane to the spatial light modulator (SLM) plane, which is usually referred as “I-to-S” transforming. In DWDH printers, there are six principal planes, i.e., the hologram plane, the SLM plane, the projected SLM image plane, the camera plane, the film plane and the projected film plane. There is the assumption of a small-aperture camera and a “point” hogel, and each image of the SLM plane is acquired by the pixel swapping technique from different images of film planes according to the ray-tracing principle. Consequently, there is an exact pixel matching relationship between the SLM plane and film plane. Moreover, with the development of computer graphic, the image data for DWDH stereograms can be acquired rapidly from a 3D digital model with a double-frustrum camera algorithm^35,36^.

In addition to the methods above, a novel method based on effective perspective images’ segmentation and mosaicking (EPISM) was proposed by our group^37^. On the basis of ray-tracing principle and the reversibility of light propagation, the viewing frustum effect of human eyes was utilized. With the segmentation and mosaicking of effective images, synthetic effective perspective images for single-step exposure could be achieved. In nature, the proposed method imitated and modified the reproduction of master hologram to transfer hologram in two-step method. There are only three principal planes in EPISM method, i.e., the virtual master hologram plane, the LCD plane, and the transfer hologram plane.

In our opinion, the infinite viewpoint camera method, the single-step Lippmann holographic stereogram method and the DWDH method can all be classified as the method of single pixel mapping, whereas the EPISM belongs to the method of image block operation. Although the EPISM method is also based on the ray-tracing principle, it is different from the DWDH method. The camera plane and the hologram plane in DWDH method can be considered equally to the virtual master hologram plane and the transfer hologram plane in EPISM method correspondingly. In DWDH method, hogels in the camera plane and the hologram plane are both assumed as “point” hogels, and the pixel matching relationship is accurate. Each image of film plane contributes the equal numbers of pixels to the image of SLM plane. Once the parameters of the printing system (such as the distance between the camera plane and the hologram plane, the pixel intervals of film plane and SLM plane) are determined, the relationship between the hogel sizes of the camera plane and the hologram plane are fixed. However, in our proposed EPISM method, only the hogels in transfer hologram are considered as “point” hogels. The images for hogels in transfer holograms are acquired by the pixel mosaicking technique, not the pixel swapping technique. Each image of the virtual hogel in virtual master hologram contributes different numbers of pixels to the image of the hogel in transfer hologram, and the calculation burden in EPISM method is much less than that of the DWDH method, especially for a full parallax holographic stereogram. Moreover, since it is the pixel mosaicking technique, the hogel sizes in virtual master hologram and transfer hologram can be arbitrary, without any limitations.

The optimization of system parameters and the evaluation of imaging quality are the focuses of research in holographic stereogram printing, and the optical transfer function (OTF) is an efficient tool to evaluate the imaging quality of holographic stereogram from the aspect of frequency response^38^. St.-Hilaire constructed the modulation transfer function (MTF) of horizontal-parallax-only (HPO) image-plane holographic stereograms, and discussed the optimum sampling of the slit plane with fixed depth object points^39–41^. Helseth investigated the OTF of 3D display systems, and considered the influence of the Stiles-Crawford effect on human eyes^42^. In our previous study, the reconstructed wavefront errors in holographic stereogram were expressed as defocusing aberrations, and the frequency responses of full parallax holographic stereogram were studied when the rectangle, the shaped Gaussian, and the shaped Blackman window functions were used as the exit pupil functions respectively, then the design criterion of the exit pupil function was also discussed^43^.

However, the researches mentioned above are all applied to the situation when there is only a single hologram considered. In EPISM based holographic stereogram printing method, the function model is more complicated, since the imaging quality of holographic stereogram depends on not only the hogel in transfer hologram, but also the virtual hogel in master hologram. In view of the EPISM based holographic stereogram, the function model of the printing system is established, and the OTF is investigated. According to the reproduction effect of the 3D scene, the influence of the exit pupil size on the frequency response of holographic stereogram is analyzed in detail. Moreover, by considering the flipping effect in spatial domain, and considering the complete transitivity of the image information during the simulated reproduction process of master hologram to transfer hologram, the value of hogel size is optimized.

## Methods

### Exit pupil function of EPISM based holographic stereogram

**Figure 1:**
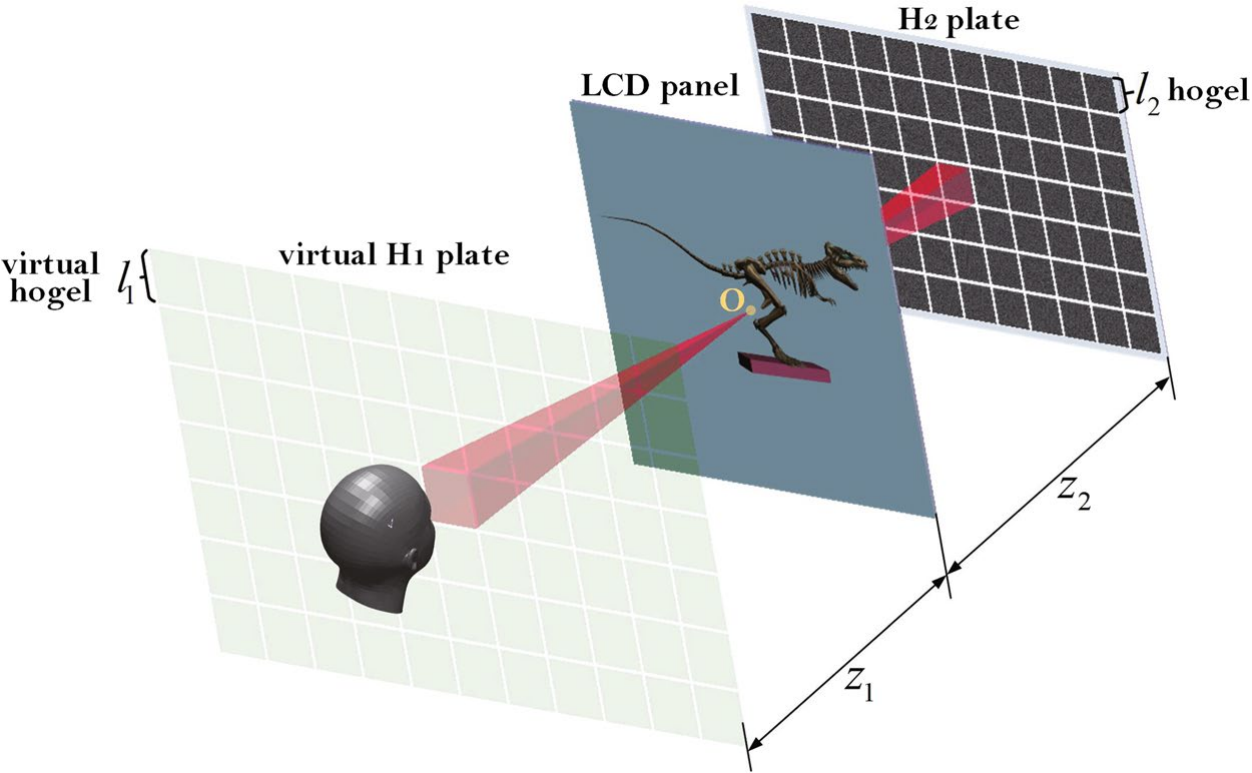
The exit pupil function model of EPISM based holographic stereogram.

The exit pupil function model of EPISM based holographic stereogram is shown in Fig. 1. The virtual H_1_ plate, LCD panel and H_2_ plate are placed parallelly along the *z*-axis, and the distances among them are *z*_1_ and *z*_2_, respectively. Point O on LCD panel is located on the center of hogels in virtual H_1_ plate and H_2_ plate, and light rays emitted by point O are diffused to the hogels with frustum patterns. The diameter of human pupil is assumed as about *l*_*e*_ = 3~5 mm. The virtual hogels in virtual H_1_ plate and the hogels in H_2_ plate are all with square and hard exit pupils, whose sizes are *l*_1_ and *l*_2_ respectively, and they are supposed to be smaller than the size of human pupil, i.e., *l*_*e*_ > *l*_1_ and *l*_*e*_ > *l*_2_.

**Figure 2:**
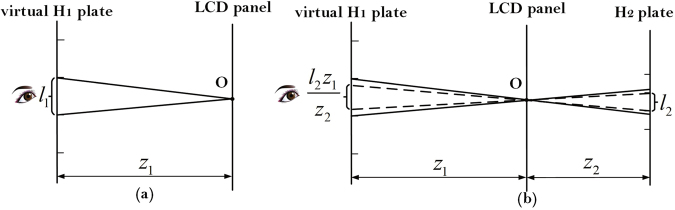
The simplified or modified exit pupil function models under two different conditions. (**a**) When ${l}_{2}\ge \frac{{l}_{1}{z}_{2}}{{z}_{1}}$; (**b**) when ${l}_{2} < \frac{{l}_{1}{z}_{2}}{{z}_{1}}$.

For most holographic stereograms, there is some distance between the viewing plane and the exit pupil plane (virtual H_1_ plate). For simplicity, the viewing plane is set at the exit pupil plane, and only one-dimension case is analyzed. The deduced conclusions can be extended to two-dimension case easily. Exit pupil is a virtual aperture which confines the light rays of an optical system, i.e., only light rays passing through the exit pupil can exit the system and enter the human eyes. Since the light rays emitted by the point on LCD panel are both discretely recorded by virtual H_1_ plate and H_2_ plate, when ${l}_{2}\ge \frac{{l}_{1}{z}_{2}}{{z}_{1}}$, the virtual hogel in virtual H_1_ plate determines the exit pupil, and the exit pupil function model is simplified as shown in Fig. 2(a). On the contrary, when ${l}_{2} < \frac{{l}_{1}{z}_{2}}{{z}_{1}}$, the exit pupil is determined by the hogel in H_2_ plate, and the exit pupil function model is modified as shown in Fig. 2(b).

Consequently, the exit pupil function model of EPISM based holographic stereogram can be shown uniformly in Fig. 2(a), and the exit pupil size *l* is expressed as

1 $$l=\{\begin{array}{ll}{l}_{1} & {\rm{when}}\,{l}_{2}\ge \frac{{l}_{1}{z}_{2}}{{z}_{1}}\\ \frac{{l}_{2}{z}_{1}}{{z}_{2}} & {\rm{when}}\,{l}_{2} < \frac{{l}_{1}{z}_{2}}{{z}_{1}}.\end{array}$$

### Optical transfer function with defocusing aberrations

In a conventional hologram, the wavefront of the 3D scene is reconstructed by the complete amplitude and phase information, where the amplitude and phase present the luminance and depth of any object point respectively. However, in the holographic stereogram, the accurate phase information isn’t recorded when achieving a sequence of two-dimensional perspective images, as a series of wavefront segments are used to approximate the true wavefront of the 3D scene^44^. Many researchers are studying the accommodation cues and continuous motion parallax in holographic stereogram, and have proposed many solutions to solve the defocusing errors, such as reconfigurable image projection (RIP) algorithm^45,46^ and diffraction specific coherent panoramagram (DSCP) algorithm^47^. For simplification of analysis in our manuscript, when observing the holographic stereogram, the perceived curvature radius of wavefront is only a measurement of the distance between the LCD panel and the observer. Obviously, there will be reconstructed wavefront errors during the hologram reconstruction when the object points aren’t located at the LCD panel, and they are mainly expressed as defocusing aberrations. The defocusing aberrations will lead to the decline of amplitude and the variation of phase of the wavefront, reducing the imaging quality.

**Figure 3:**
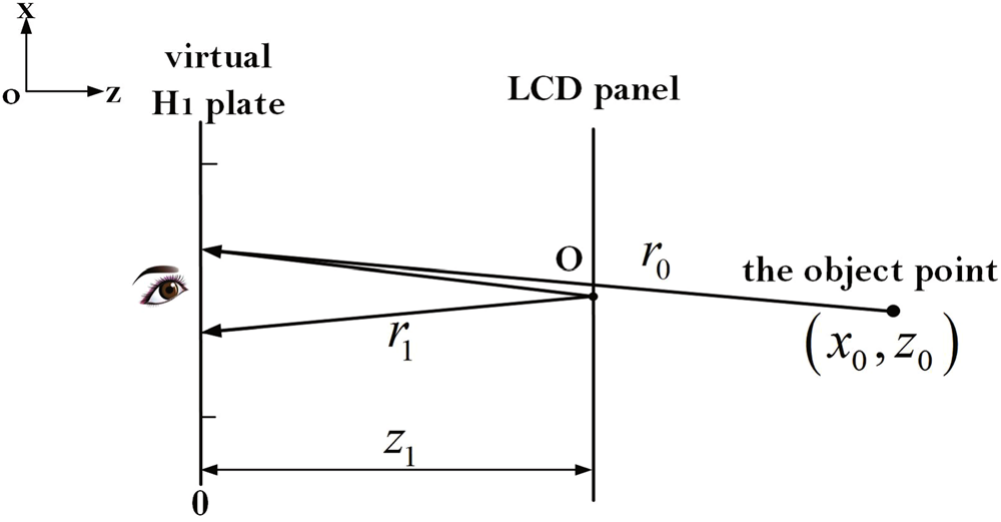
The reconstructed wavefront error in holographic stereogram (the viewing plane is coincident with the virtual H_1_ plane).

For an isolated point in 3D space, its wavefront can be considered as a spherical pattern, and the reconstructed wavefront error with respect to the original scene depth is shown in Fig. 3. The object point is assumed to be at the position (*x*_0_, *z*_0_) in space, and the observer at the center of virtual hogel in virtual H_1_ plate will locate the point at (*x*_0_, *z*_1_), where *x*_0_ is the central coordinate of the exit pupil in the viewing plane. The origin point of *z*-axis is fixed at the viewing plane.

When the pixel of point O on LCD panel is diffused to the exit pupil, with a Fresnel approximation, the curvature error of wavefront can be expressed as

2 $$W(x)={r}_{0}-{r}_{1}\approx {z}_{0}-{z}_{1}+\frac{{z}_{1}-{z}_{0}}{2{z}_{0}{z}_{1}}{(x-{x}_{0})}^{2}\mathrm{.}$$

It is supposed that *x*_0_ = 0, and the first term *z*_0_ − *z*_1_ will be omitted in the future analysis as it only adds a constant phase factor.

Then the defocusing aberrations can be expressed as

3 $$\exp [{\rm{j}}kW(x)]=\exp ({\rm{j}}k\frac{{z}_{1}-{z}_{0}}{2{z}_{0}{z}_{1}}{x}^{2}),$$

where *k* = 2/*λ* is the wavenumber and *λ* is the wavelength of the laser source.

For an optics system with a square and hard exit pupil, the generalized exit pupil function **P**(*x*) can be written as

4 $${\bf{P}}({x})=P(x)\exp [{\rm{j}}kW(x)],$$

where $P(x)={\rm{rect}}(x/l)$, is the rectangle window function. The generalized exit pupil function **P**(*x*) contains not only the limitations of the size and the shape of the exit pupil, but also the function of the system aberrations.

The diffraction limited system can be usually regarded as a linear shift-invariant system. With the knowledge of Fourier transform (FT) and the point spread function (PSF), the impulse response of the system is proportional to the FT of the exit pupil function. Then the coherent transfer function ${\bf{P}}(\lambda {z}_{1}{f}_{x})$ of the system can be expressed as

5 $${\bf{P}}(\lambda {z}_{1}{f}_{x})=P(\lambda {z}_{1}{f}_{x})\exp [{\rm{j}}kW({\rm{\lambda }}{z}_{1}{f}_{x})\mathrm{.}$$

The optical transfer function OTF (*f*_*x*_) can be calculated by the normalized autocorrelation function of its coherent transfer function, and it has been sufficiently studied by Goodman^48^. The result is shown as follows,

6 $${\rm{OTF}}({f}_{x})={\rm{\Lambda }}(\frac{\lambda {z}_{1}|\,{f}_{x}|}{l})\times {\rm{sinc}}[\frac{({z}_{1}-{z}_{0})}{{z}_{0}{z}_{1}}\cdot (l{z}_{1}{f}_{x})\cdot (1-\frac{\lambda {z}_{1}|{f}_{x}|}{l})],$$

where sinc(*x*) = sin(π*x*)/π*x*, and the triangular function is defined as

7 $${\rm{\Lambda }}(x)=\{\begin{array}{ll}1-|x| & |x|\le 1\\ 0 & {\rm{else}}{\rm{.}}\end{array}$$

Similarly, the OTF of a full parallax holographic stereogram is

8 $$\begin{array}{c}{\rm{OTF}}({f}_{x},{f}_{y})={\rm{\Lambda }}(\frac{\lambda {z}_{1}|\,{f}_{x}|}{l})\Lambda (\frac{\lambda {z}_{1}|\,{f}_{y}|}{l})\times {\rm{sinc}}[\frac{({z}_{1}-{z}_{0})}{{z}_{0}{z}_{1}}(l{z}_{1}\,{f}_{x})(1-\frac{\lambda {z}_{1}|\,{f}_{x}|}{l})]\\ \quad \quad \quad \quad \quad \quad \times \,{\rm{sinc}}[\frac{({z}_{1}-{z}_{0})}{{z}_{0}{z}_{1}}(l{z}_{1}{f}_{y})(1-\frac{\lambda {z}_{1}|\,{f}_{y}|}{l})]\mathrm{.}\end{array}$$

**Figure 4:**
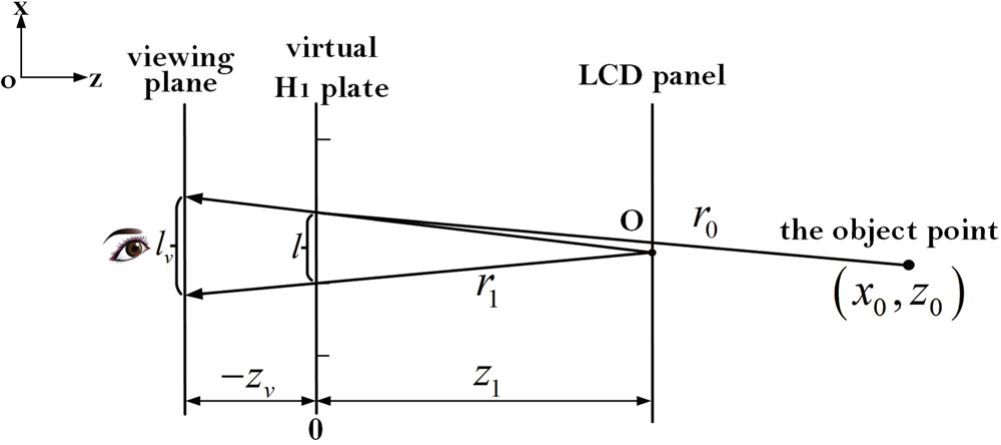
The reconstructed wavefront error in holographic stereogram (the viewing plane isn’t coincident with the virtual H_1_ plane).

Moreover, when the viewing plane is not coincident with the virtual H_1_ plane, we suppose the coordinate of viewing plane is *z*_*v*_ on the z-axis (*z*_*v*_ < 0). Then the reconstructed wavefront error in holographic stereogram is shown in Fig. 4.

The value of *l*_*v*_ is expressed as ${l}_{v}=\frac{{z}_{0}-{z}_{v}}{{z}_{0}}\times l$, where *l* is shown in Eq. (1). If *l*_*v*_ ≥ *l*_*e*_, the exit pupil size of the system is *l*_*e*_, otherwise, the exit pupil size of the system is *l*_*v*_. Then the OTF of a full parallax holographic stereogram can be expressed as

9 $$\begin{array}{l}\begin{array}{c}{\rm{OTF}}({f}_{x},{f}_{y})={\rm{\Lambda }}[\frac{\lambda ({z}_{1}-{z}_{v})|{f}_{x}|}{l^{\prime} }]{\rm{\Lambda }}[\frac{\lambda ({z}_{1}-{z}_{v})|{f}_{y}|}{l^{\prime} }]\\ \quad \quad \quad \quad \quad \times \,{\rm{sinc}}\{\frac{({z}_{1}-{z}_{0})}{{z}_{0}({z}_{1}-{z}_{v})}[l^{\prime} ({z}_{1}-{z}_{v}){f}_{x}][1-\frac{\lambda ({z}_{1}-{z}_{v})|{f}_{x}|}{l^{\prime} }]\}\end{array}\\ \quad \quad \quad \quad \quad \times \,{\rm{sinc}}\{\frac{({z}_{1}-{z}_{0})}{{z}_{0}({z}_{1}-{z}_{v})}[l^{\prime} ({z}_{1}-{z}_{v}){f}_{y}]\,[1-\frac{\lambda ({z}_{1}-{z}_{v})|{f}_{y}|}{l^{\prime} }]\},\end{array}$$

where $$l^{\prime} =\{\begin{array}{ll}{l}_{e} & {\rm{when}}\,\,{l}_{v}\ge {l}_{e}\\ {l}_{v} & {\rm{when}}\,\,{l}_{v} < {l}_{e}.\end{array}$$

For simplicity, all the experiments and analyses in the following sections are all based on the situation depicted in Fig. 3, and Eq. (8) is utilized.

## Results and Discussions

### Numerical simulations

From the analysis mentioned above, there are defocusing aberrations during the reconstruction of holographic stereogram, and the defocusing aberration is determined by the distance between the object point in space and the LCD panel, and the farther the distance is, the greater the aberration will be. To illustrate the influence of exit pupil size on the OTF, the parameters of printing system in our previous work^37^ are taken as an example. The field of view (FOV) is supposed as θ = 30°, and the corresponding parameters are *z*_0_ = 186 mm and λ = 639 nm. The LCD panel is located at the mid-plane of the 3D scene. The depth distribution of the 3D scene is about Δ*z* = −50 mm ~50 mm, where it is supposed that Δ*z* = 0 at the LCD panel. For different aberration conditions, the optimized sizes of exit pupil functions will not be the same.

**Figure 5:**
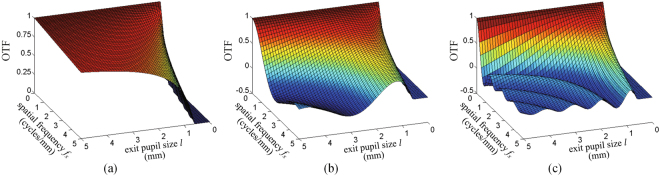
The values of OTF with respect to the spatial frequency *f*_*x*_ and the exit pupil size *l* under different aberration conditions. (**a**) Non-aberration plane; (**b**) middle aberration plane; (**c**) high aberration plane.

We choose three different planes, which are non-aberration plane at Δ*z* = 0 mm, middle aberration plane at Δ*z* = −20 mm and high aberration plane at Δ*z* = −50 mm, and calculate the OTF of each plane against exit pupil size and spatial frequency. The results are shown in Fig. 5. For simplicity, the results only show the variations of OTF based on spatial frequency *f*_*x*_ and exit pupil size *l*, and they are only the cases of positive spatial frequency, i.e., *f*_*x*_ > 0.

The max spatial frequency is set as 5 cycles/mm according to the characteristic of the LCD panel we used. In our experiment, a LCD panel (VVX09F035M20) produced by Panasonic is used. It is 8.9 inches with 1920 × 1080 pixels, and the pixel interval is about Δ*l* = 0.1 mm. Therefore, the maximum spatial frequency of image displayed by the LCD panel is calculated as ${f}_{x{\rm{\max }}}={f}_{y{\rm{\max }}}=\frac{1}{2{\rm{\Delta }}l}$ = 5 cycles/mm.

As shown in Fig. 5, the OTF drops towards zero rapidly at relatively small spatial frequency for very small values of *l*. When there is a defocusing aberration, the OTF decreases more quickly, and there occurs oscillations on the edges, and the value of OTF turns to be negative. That is to say, the sign reversal of the OTF occurs, which means the contrast reversal, and will reduce the imaging quality seriously. Meanwhile, the greater the defocusing aberration is, the more rapid the descent and the more obvious the oscillations of the OTF will be, which will lead to a reduction of reconstruction quality.

For a certain defocusing aberration of the 3D scene plane, to investigate the global behavior of full parallax holographic stereogram on all spatial frequencies, the optimized exit pupil size is determined by the corresponding OTF which has the highest average integral value across the passband, which can be calculated as

10 $$\Upsilon =\frac{{\int }_{0}^{{f}_{x{\rm{\max }}}}{\int }_{0}^{{f}_{y{\rm{\max }}}}{\rm{OTF}}({f}_{x},{f}_{y}){\rm{d}}{f}_{x}{\rm{d}}{f}_{y}}{{\int }_{0}^{{f}_{x{\rm{\max }}}}{\int }_{0}^{{f}_{x{\rm{\max }}}}{\rm{d}}{f}_{x}{\rm{d}}{f}_{y}}\mathrm{.}$$

**Figure 6:**
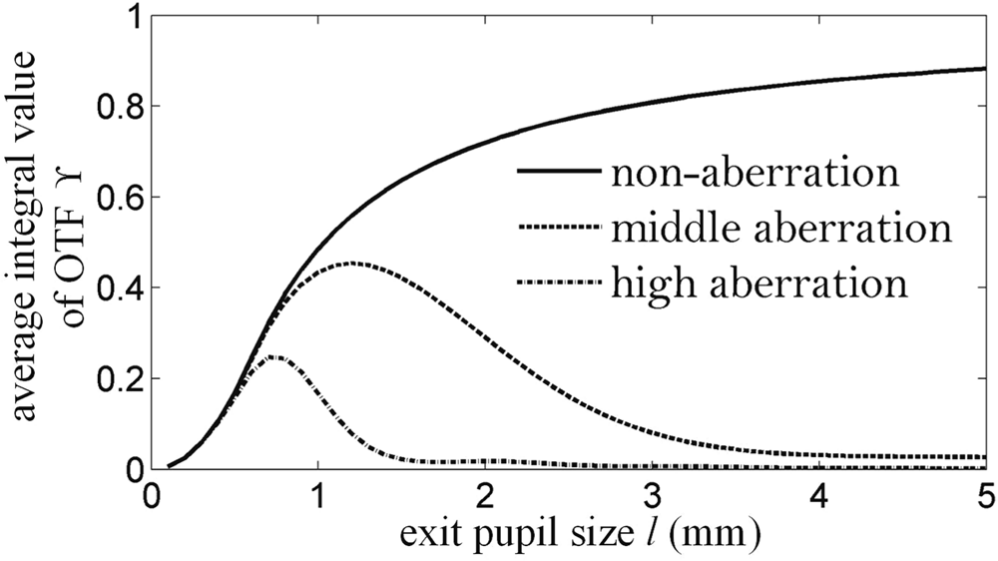
The average integral values of OTF ϒ with respect to the exit pupil size *l* under different aberration conditions.

A larger ϒ means a higher modulated ability for the whole passband. Considering the holographic stereogram printing system which we have given, the variations of ϒ with respect to exit pupil size in different aberration planes are shown in Fig. 6.

As shown in Fig. 6, the optimized exit pupil sizes *l*_opt_ of holographic stereograms with non-aberration, middle aberration and high aberration are 5 mm, 1.2 mm and 0.7 mm, respectively. A larger exit pupil size is better when there is no aberration, and the holographic stereogram has the highest average integral value of the OTF. Because of the limited human pupil size, the maximum exit pupil size cannot exceed 5 mm. For the plane with aberration, the greater the aberration is, the smaller the optimized exit pupil size will be.

**Figure 7:**
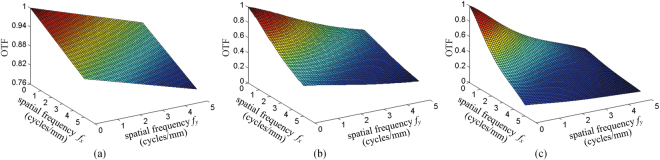
The values of OTF with the optimized exit pupil sizes *l*_opt_ under different aberration conditions. (**a**) Non-aberration plane, *l*_opt_ = 5 mm; (**b**) middle aberration plane, *l*_opt_ = 1.2 mm; (**c**) high aberration plane, *l*_opt_ = 0.7 mm.

The behavior of OTF with the optimized exit pupil sizes *l*_opt_ under different aberration conditions are shown in Fig. 7. Obviously, the greater the aberration is, the more rapidly the OTF decreases, and there are no oscillations occoured in Fig. 7 as the optimized exit pupil sizes are used under different aberration conditions.

**Figure 8:**
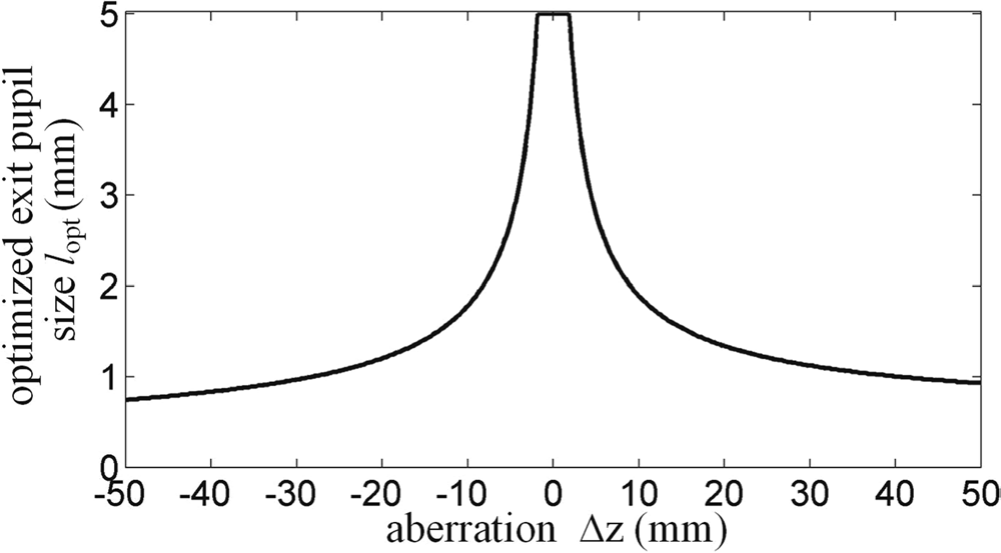
The optimized exit pupil size *l*_opt_ with respect to the aberration plane, Δ*z* = −50 mm ~50 mm.

For the more general case, optimized exit pupil size under different aberration conditions are given in Fig. 8, and Δ*z* is changing from −50 mm to 50 mm. As shown in Fig. 7, for the same 3D scene depth (the absolute values of aberrations are identical), the optimized exit pupil sizes are slightly different when the value of aberration is positive or negative. However, the variation trend is consistent, namely, the higher the 3D scene plane deviating from the LCD panel (zero error plane) is, the smaller the corresponding optimized exit pupil size will be.

**Figure 9:**
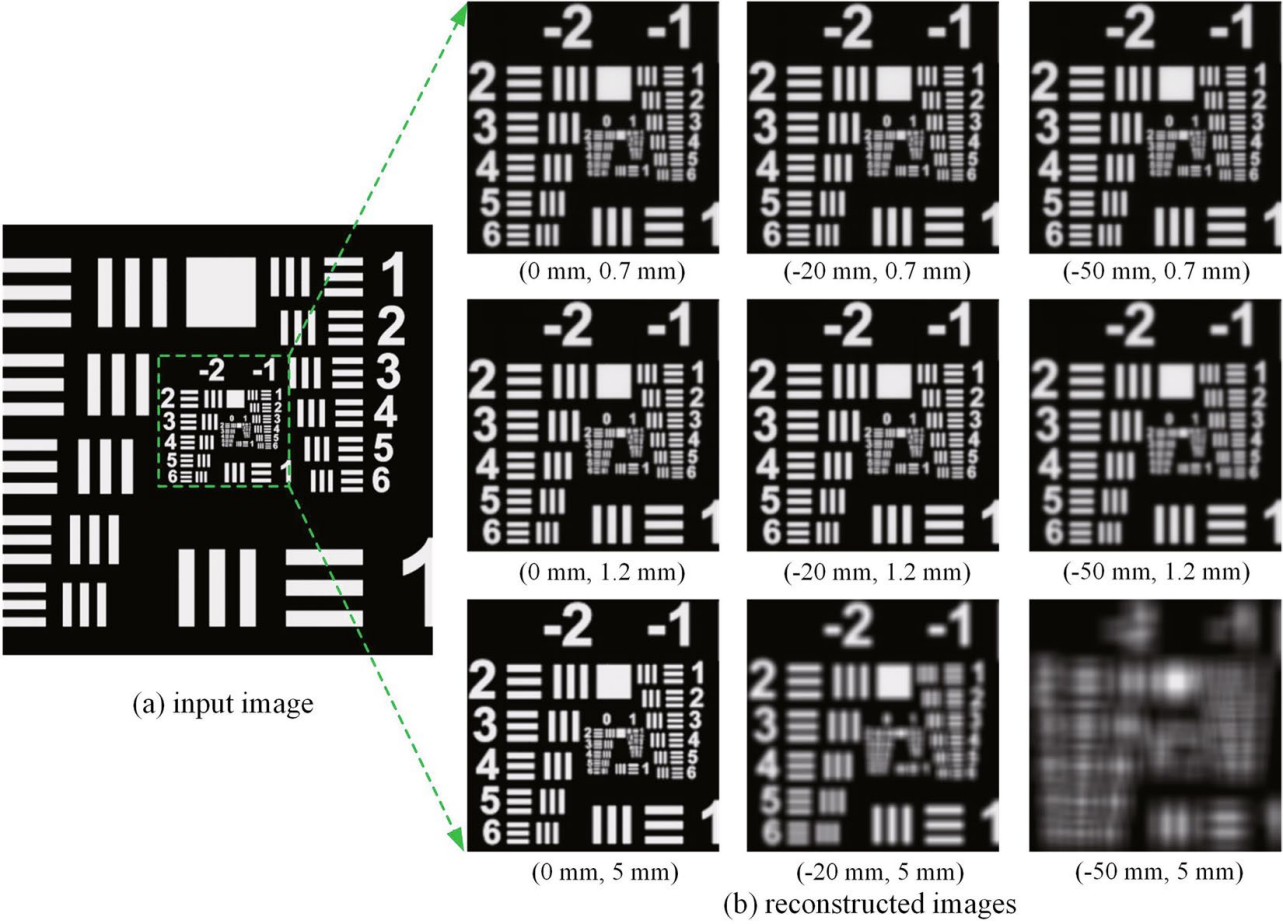
Degradation of the imaging quality for the reconstructed images under different aberration conditions Δ*z* and different exit pupil sizes *l*, (Δ*z*, *l*).

In order to verify the relationship between the optimized exit pupil size and the aberration, we use single perspective image of a resolution test target model as the original input image to conduct the simulation. For the three cases of non-aberration plane, middle aberration plane and high aberration plane, after passing through different pupils, the reconstructed images are shown in Fig. 9. The values of peak signal to noise ratio (PSRN) of different reconstructed images are also calculated as shown in Table 1.

**Table 1:** PSRN of different reconstructed images with respect to the aberration and exit pupil size.

Exit pupil size (mm)	Aberration (mm)		
	0	−20	−50
0.7	27.98	27.73	25.95
1.2	32.55	30.69	23.48
5	44.72	20.42	13.13

It can be seen that the simulation results are consistent with the theoretical analyses above. The aberrations will degrade the imaging quality, and the greater the aberration is, the worse the imaging quality will be. The optimized exit pupil size is 5 mm when there is no aberration, and the value of PSRN is the highest, while the optimized exit pupil sizes are 1.2 mm and 0.7 mm in middle-aberration plane and high-aberration plane respectively.

From the analysis above, we can get the optimized exit pupil size corresponding to a certain depth plane of the 3D scene. When considering the complete 3D scene, the variation of aberration is added to the evaluation index ϒ_tot_, namely, the depth of the 3D scene is also considered to be integrated. A larger ϒ_tot_ means a higher modulated ability for the whole passband of the complete 3D scene.

11 $${\Upsilon }_{{\rm{tot}}}=\frac{{\int }_{{z}_{1}-{\rm{\Delta }}z}^{{z}_{1}+{\rm{\Delta }}z}{\int }_{0}^{{f}_{x{\rm{\max }}}}{\int }_{0}^{{f}_{y{\rm{\max }}}}{\rm{OTF}}({f}_{x},{f}_{y}){\rm{d}}{f}_{x}{\rm{d}}{f}_{y}{\rm{d}}{z}_{0}}{{\int }_{{z}_{1}-{\rm{\Delta }}z}^{{z}_{1}+{\rm{\Delta }}z}{\int }_{0}^{{f}_{x{\rm{\max }}}}{\int }_{0}^{{f}_{y{\rm{\max }}}}{\rm{d}}{f}_{x}{\rm{d}}{f}_{y}{\rm{d}}{z}_{0}}\mathrm{.}$$

**Figure 10:**
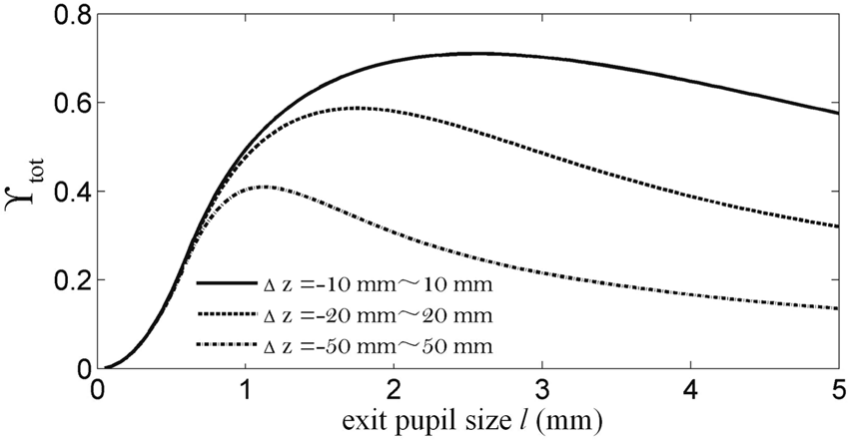
ϒ_tot_ with respect to the exit pupil size *l* under different depth distributions of the 3D scene.

The variations of ϒ_tot_ with respect to exit pupil size in different depth distributions of the 3D scene are shown in Fig. 10. The optimized values of exit pupil size are 2.55 mm, 1.75 mm and 1.1 mm respectively for 3D scenes with different depths. The results indicate that there is an optimized value of the exit pupil size with a certain depth distribution of the 3D scene, and this optimized value is smaller when the depth distribution of the 3D scene is broader.

### Optical experiments

**Figure 11:**
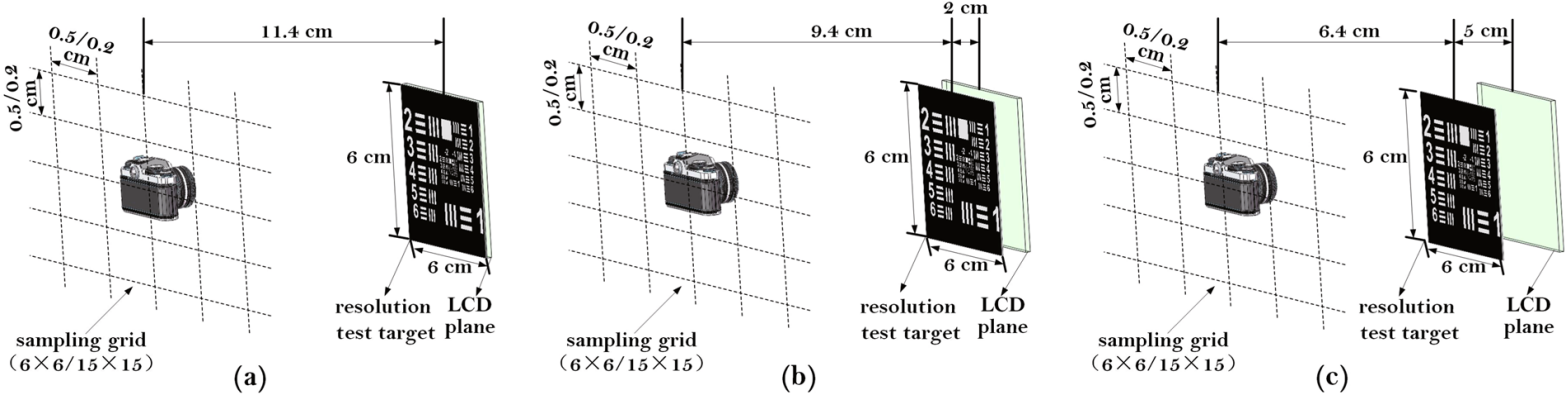
The diagram of experimental model with resolution test target under different aberration conditions. (**a**) Δ*z* = 0 mm; (**b**) Δ*z* = −20 mm; (**c**) Δ*z* = −50 mm.

To demonstrate the validity of our analysis in numerical simulations when the reconstructed image is under a certain aberration condition, an optical experiment is designed. The experimental model with resolution test target under different aberration conditions is shown in Fig. 11. The distance between the camera sampling plane and the LCD panel is 11.4 cm, while the resolution test target plane is 0 cm, 2 cm, or 5 cm distant from the LCD panel, to express non-aberration aberration, middle aberration or high conditions, respectively. The sampling grid is 6 × 6 or 15 × 15, while the sampling interval is 0.5 cm or 0.2 cm correspondingly. The resolution of sampling perspective images is 600 pixel × 600 pixel. The hogel sizes of holographic stereogram printing are 0.5 cm or 0.2 cm in practice.

**Figure 12:**
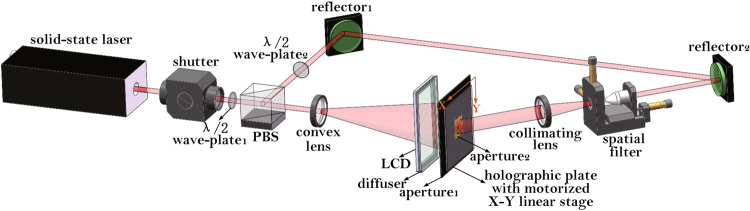
Optical setup of holographic stereogram printing system.

The optical setup of the holographic stereogram printing system is shown in Fig. 12. A 400 mW 639 nm single longitudinal mode and linear polarization solid-state red laser (CNI MSL-FN-639) is used as the laser source, and an electric shutter (Sigma Koki SSH-C2B) is used to control the exposure time. The laser beam passes a λ/2 wave-plate and a polarizing beam splitter (PBS), then divides into two beams, i.e., the signal beam and the reference beam. Intensity ratio of the signal beam and the reference beam is adjusted by the first λ/2 wave-plate, and the other λ/2 waveplate is used to adjust the polarization state of the reference beam, to keep the polarization state consistent between the two beams. On the signal beam path, a series of images are displayed on the LCD panel with a diffuser, then the light rays with image information are diffused onto the holographic plate. Holographic plate is sandwiched between two apertures to ensure only a square area of the holographic plate (i.e., the hogel) exposed, and it is installed on a motorized KSA300 X-Y stage which is driven by a programmable MC600 controller. The distance between the LCD panel and the holographic plate is 11.4 cm. The reference beam passes through a spatial filter comprised of a 40× objective and a 15 μm pin-hole to filter out the higher spatial frequency, then modulated by a collimating lens to get a uniform plane wave. The reference beam is about 40° off from the normal axis of the holographic plate. The signal beam and the reference beam are interfered from different sides, and the interference fringes are recorded on the holographic plate.

In our EPISM based holographic stereogram printing system, we don’t adopt a more usual printer configuration with a FT high numerical aperture (NA) lens system which is referred to as a lens-based printer. The reasons are as follows. When utilizing the NA objective lens, the FOV of the hologram will be fixed, however the value of FOV can be variable in EPISM based holographic stereogram. Moreover, the objective lens will bring in the image distortion more or less. It is such a principle verification experiment that we don’t care much about the printing efficiency, so a LCD panel and a diffuser are used for printing, not a lens-based configuration. Nevertheless, the EPISM based holographic stereogram can be also applied to the lens-based printers, as long as the field angle between the real image of SLM and the hogel keeps the same with the field angle between the LCD panel and the hogel.

**Figure 13:**
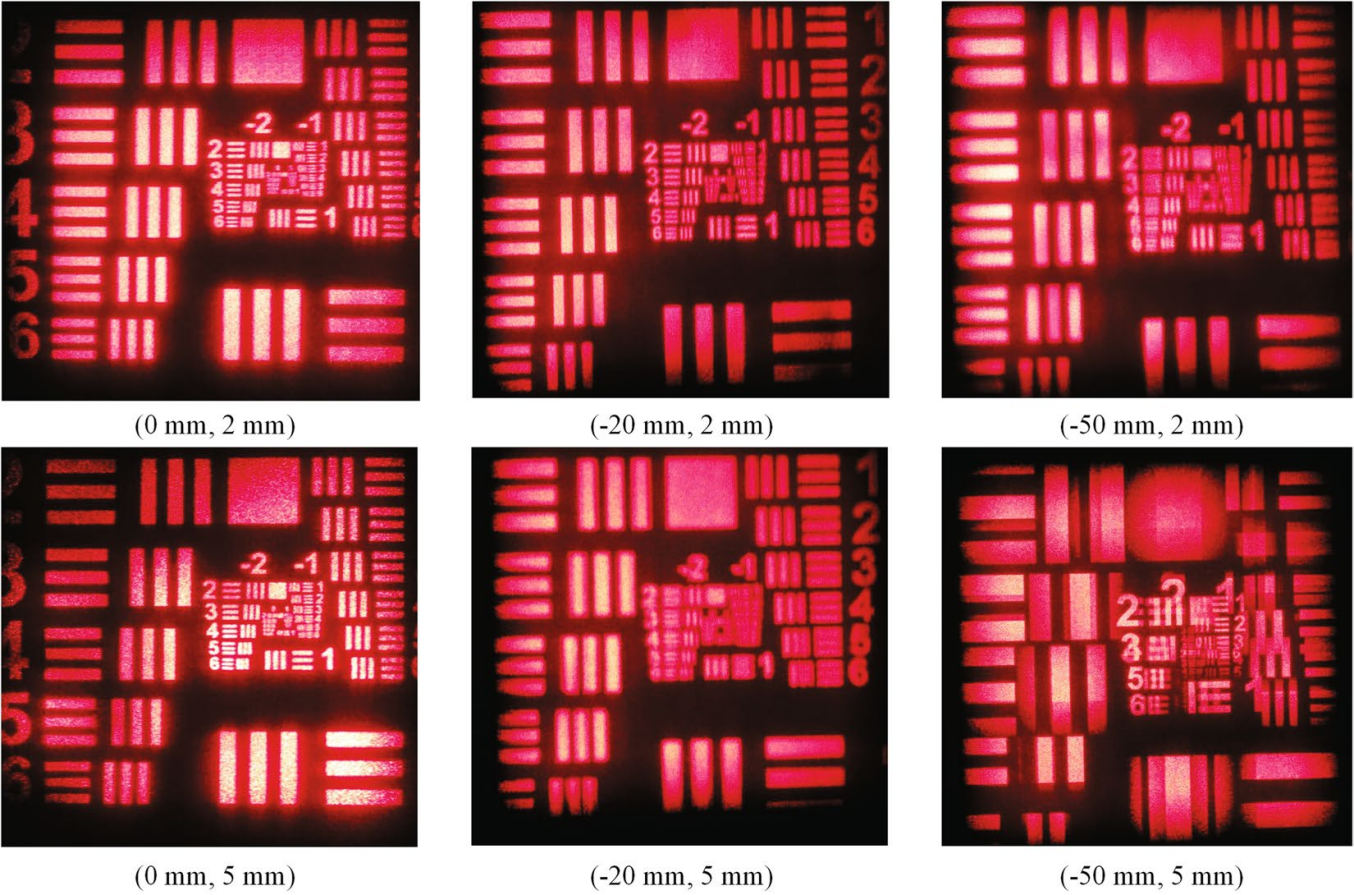
The images of optical reconstruction under different aberration conditions Δ*z* with different exit pupil sizes *l*, (Δ*z*, *l*).

A Canon EOS 5D camera with a 100-mm focus lens is put about 40 cm in front of the holographic plate to capture the reconstructed images. Optical reconstruction images are shown in Fig. 13 under different aberration conditions, and they are all virtual images inside of the holographic plate.

As shown in Fig. 13, when it is under the non-aberration condition, the reconstructed effects are both well whether the hogel size is 2 mm or 5 mm. When there exists an aberration, the reconstructed effect with 2 mm hogel size is much better than that of 5 mm hogel size, especially under a higher aberration condition. If the aberration is −50 mm and the hogel size is 5 mm, the effect is so bad that we can hardly focus on the reconstructed image when capturing photos. It should be pointed out that, since the camera with a macro lens is used, the view field size could be captured by the camera lens is limited. When the aberration is higher, the reconstructed image is closer to the camera, so the range of the image captured by the camera is smaller.

**Figure 14:**
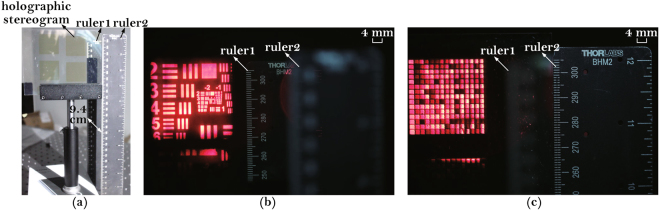
The images captured at different focus depths. (**a**) The spatial position relation of rulers and holographic plate; (**b**) focused on ruler1; (**c**) focused on ruler2.

Take the middle aberration condition as an example, to express the location of reconstructed image. One ruler is placed parallel to the holographic plate, and the other one is placed 9.4 cm behind. The images captured at different focus depths are shown in Fig. 14. The spatial position relation is shown in Fig. 14(a). In Fig. 14(b), both the reconstructed image and ruler1 are clear simultaneously while ruler2 is blurred. In Fig. 14(c), both the holographic plate and ruler2 are clear simultaneously, and the printed hogels can be observed clearly, while ruler1 is blurred.

We have discussed the effect of the exit pupil size on the imaging quality from the aspect of frequency domain above. Now we will take the spatial domain analysis into consideration, and consider whether the information can transmit completely during the simulated reproduction process of master hologram (virtual H_1_ plate) to transfer hologram (H_2_ plate), to find the optimized hogel sizes for EPISM based holographic stereogram printing system, i.e., the optimized size of virtual hogel (*l*_1_) in virtual H_1_ plate and the optimized size (*l*_2_) of hogel in H_2_ plate. Based on the depth distribution of the 3D scene, and the virtual or real reconstructed effect finally, we can determine the parameter *z*_2_ of the EPISM based holographic stereogram printing system.

Considering the flipping effect when observing the holographic stereogram, Yatagai analyzed the maximum value of 3D scene depth range Δ*z*_range_ in spatial domain as^49^

12 $${\rm{\Delta }}{z}_{{\rm{range}}}\approx \frac{2.44{\rm{\lambda }}{z}^{2}}{{D}^{2}},$$

where *D* denotes the hogel size, and *z* denotes the distance between the hologram plane and the image plane.

Taking Eq. (12) into EPISM based holographic stereogram printing system, we have the condition that

13 $${l}_{1}\le =\sqrt{\frac{2.44{\rm{\lambda }}{{z}_{1}}^{2}}{{\rm{\Delta }}{z}_{{\rm{range}}}}}\mathrm{.}$$

As the light rays emitted by any point in LCD are recorded discretely by both the virtual H_1_ plate and H_2_ plate, and at the center of each virtual hogel or hogel, the printing system should satisfy the condition of ${l}_{2} < \frac{{l}_{1}{z}_{2}}{{z}_{1}}$ to ensure that there is no information lost during the reproduction. Consequently, the exit pupil is determined by the hogel in H_2_ plate as shown in Fig. 2. Furthermore, as shown in Fig. 10, when the depth distribution of the 3D scene is determined, the hogel size can be optimized from the frequency domain analysis to achieve a higher modulated ability of the hologram.

More specifically, the reconstructed 3D scene is supposed to be 114 mm outside of the holographic medium, and the parameters *z*_1_ = 186 mm, *z*_1_ = 114 mm, λ = 639 nm and Δ*z*_range_ = 2 × 10 = 20 mm are taken into equations above, finally the optimized hogel sizes for EPISM based holographic stereogram printing system are

14 $$\{\begin{array}{l}{l}_{1}=\sqrt{\frac{2.44\times 639\times {186}^{2}}{{\rm{20}}}}\,=\,1.64\,{\rm{mm}}\\ {l}_{2} < \frac{114}{186}\times 1.64={\rm{1}}\,{\rm{mm}}{\rm{.}}\end{array}$$

In EPISM based holographic stereogram printing system with above conditions, the ideal value of *l*_1_ is 1.64 mm, and it represents the sampling interval of the original perspective images. However, this value is too small to resulted in a serious time cost for sampling, and it is the most extreme condition to avoid flipping effect in terms of the farthest object point deviating away from the LCD panel. During the experiment, we find that when *l*_1_ = 3.5 mm, it is enough to get a good effect for synthetic effective perspective images’ segmentation and mosaicking. Then, ${l}_{2} < \frac{{l}_{1}{z}_{2}}{{z}_{1}}$ = 2.14 mm. Referring to Fig. 10, the optimized exit pupil size is 2.55 mm. Since the exit pupil size is $l=\frac{{l}_{2}{z}_{1}}{{z}_{2}}$, then the optimized value of *l*_2_ is ${l}_{2}=\frac{l{z}_{2}}{{z}_{1}}=\frac{2.55\times 114}{186}\mathrm{=1.56}$ mm. Considering the practical situation, the value of *l*_2_ is chosen as 1.5 mm in optical holographic stereogram printing eventually.

**Figure 15:**
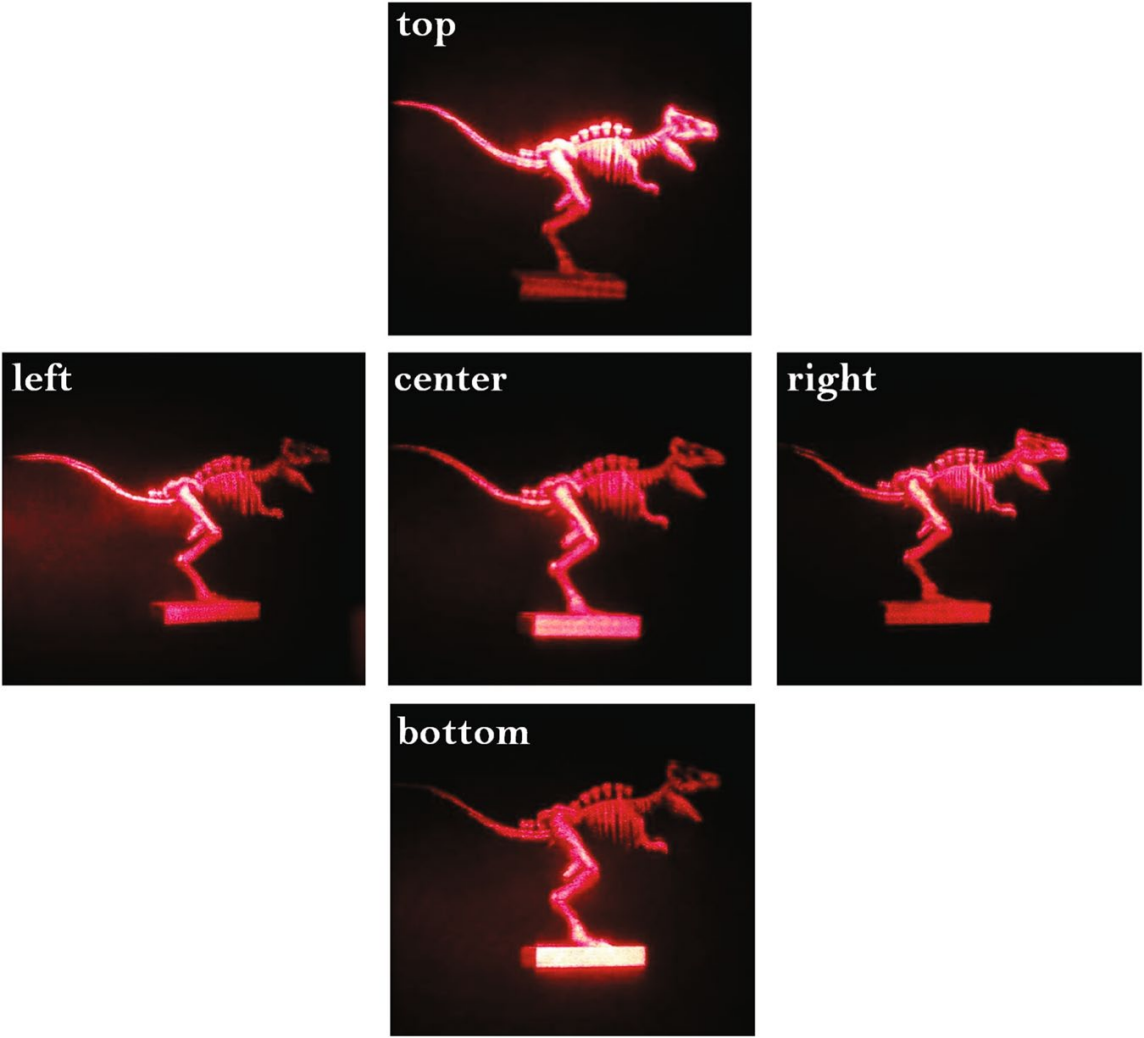
The images of optical reconstruction of a 6.2 × 2.8 × 2 cm^3^ 3D dinosaur model from different perspectives.

A dinosaur model with 6.2-cm width, 2.8-cm height and 2-cm depth is utilized as the 3D scene. The sampling interval of original perspective images is 3.5 mm, and the sampling count is 63 × 63. The printing interval of synthetic effective perspective images is 1.5 mm, and the number of hogels is 40 × 40 = 1600. The photographs of optical reconstruction from different perspectives are shown in Fig. 15, and the parallax can be reflected by the shape of the cube obviously. It can be seen that the total reconstruction quality of full parallax holographic stereogram is well when adopting the optimized parameters.

According to the analysis above, we can also get the optimized values for other parameter sets, such as different hologram sizes, different viewing distances, and different camera positions, applying the optimized procedure illustrated from Eq. (1) to Eq. (14). Therefore, the conclusions can be extended to different and more general situations.

## Conclusions

In this paper, the exit pupil function model of EPISM based holographic stereogram printing system is established, and reconstructed wavefront errors of the system are investigated and expressed as defocusing aberration. In practice, the square and hard window function is used as exit pupil function. To evaluate the imaging quality, the OTF of holographic stereogram is analyzed. Frequency responses indicate that there is an optimized value of the exit pupil size with a certain depth distribution of the 3D scene, and it is smaller when the depth distribution of the 3D scene is broader. In addition, considering the flipping effect in spatial domain, the maximum value of hogel size in virtual H_1_ plate can be achieved. Considering the complete transitivity of the image information during the simulated reproduction process of virtual H_1_ plate to H_2_ plate, the relationship between their hogel sizes is achieved. Therefore, the optimized hogel sizes in EPISM based holographic stereogram printing system can be achieved, i.e., the optimized sampling interval of original perspective images and the optimized printing interval of synthetic effective perspective images. The theoretical and experimental results indicate that the modelling agrees well with the experiments, and our OTF method will be helpful to improve the imaging quality of EPISM based holographic stereogram.
